# Sensory and chemical characteristics of *Tieguanyin* oolong tea after roasting

**DOI:** 10.1016/j.fochx.2021.100178

**Published:** 2021-12-02

**Authors:** Qing-Qing Cao, Yan-Qing Fu, Jie-Qiong Wang, Liang Zhang, Fang Wang, Jun-Feng Yin, Yong-Quan Xu

**Affiliations:** a Tea Research Institute Chinese Academy of Agricultural Sciences, Key Laboratory of Tea Biology and Resources Utilization, Ministry of Agriculture, 9 South Meiling Road, Hangzhou 310008, China; b Graduate School of Chinese Academy of Agricultural Sciences, Beijing 100081, China; c State Key Laboratory of Tea Plant Biology and Utilization, Anhui Agricultural University, Hefei, China

**Keywords:** (−)-Epigallocatechin gallate (PubChem CID: 65064), Caffeine (PubChem CID:2519), (−)-epicatechin gallate (PubChem CID: 367141), (−)-epigallocatechin (PubChem CID: 72277), (−)-epicatechin (PubChem CID: 72276), (−)-gallocatechin gallate (PubChem CID: 199,472, (−)-catechin gallate (PubChem CID: 6419835), (−)-gallocatechin (PubChem CID: 9882981), (+)-catechin (PubChem CID: 1203), Gallic acid (PubChem CID: 370), L-theanine (PubChem CID: 439378), Theobromine (PubChem CID: 5429), Roasting, *Tieguanyin* oolong tea, Sensory evaluation, Targeted analysis, Non-targeted analysis

## Abstract

Roasting, a critical process for oolong tea, has been applied to *Tieguanyin* tea to improve flavor attributes. To investigate the effects of the roasting on the flavor of *Tieguanyin*, the global metabolomics analysis on the non-volatile and volatile components were proceeded. The weakening of bitterness and astringency, caused by roasting, may be attributed to the decreasing of flavonoids glycosides and procyanidins, whereas the enhancing of sweet aftertaste to the increasing of gallic acid. Besides, l-theanine flavan-3-ols adducts (*N*-ehtyl-2-pyrrolidinone substituted flavan-3-ols) increased dramatically at 130 °C compared with 105 °C, with the reduction of l-theanine and flavan-3-ols. Meanwhile, high temperature hampered the volatiles’ diversity and intensity, resulting from the lowering of floral volatiles, i.e., β-ionone, jasmine, and nerolidol, yet the nitrogen-containing heterocyclic compounds increased, e.g., pyrroles and pyrazines. The results can help to comprehensively understand the influences of roasting technology on the flavor and chemistry of oolong tea.

## Highlights

• Roasting decreases bitterness, astringency but increases sweet aftertaste.
• Roasting degrades flavonoids glycosides and procyanidins.
• L-theanine-flavna-3-ols adducts are highly increased after roasting.
• Roasting decreases the diversity and intensity of volatile compounds.
• Pyrazines, pyrroles and furans highly increase after high temperature roasting.

## 1 Introduction

Oolong tea, one of semi-fermented teas in China, is known for its distinct floral fragrance and elegant fruit flavor (Zeng, Zhou, Su, & Yang, 2020). The unique semi-fermentation process makes the distinct quality characteristics, which are totally different from green tea (unfermented tea) and black tea (full-fermented tea). *Tieguanyin*, mainly produced in southern Fujian, is one of the most popular oolong teas around China. Similar to the other types of tea, oolong tea is rich in bioactive substances with healthy function as well, such as tea polyphenols, catechins, polysaccharides, and vitamins (Ng et al., 2018, Xu et al., 2018). Studies have found that oolong tea possess many health benefits like obesity and diabetes prevention through antioxidant and anti-inflammatory activities (Hisanaga et al., 2014, Zhang et al., 2018, Hosoda et al., 2003, Wu et al., 2018, Zhang et al., 2019).

According to aroma type, *Tieguanyin* oolong tea can be divided into light-scented and strong-scented type. Light-scented oolong tea has light and elegant floral aroma, as well as fresh and mellow flavor with sweet aftertaste. Strong-scented oolong tea has stronger fruity and roasted aroma, and it is more mellow and smooth with a stronger sweet aftertaste. The flavor differences between light-scented and strong-scented oolong tea mainly caused by the processing technology, especially the roasting procedure. Roasting is the last procedure of oolong tea processing. Light-scented oolong tea is usually slow-roasted over a low fire (i.e., 105–115 °C), while strong-scented tea is first quickly roasted at a high temperature (i.e., 130–135 °C) and then slowly roasted over a low fire. Roasting plays a crucial role during the formation of special flavor characteristics of oolong tea, especially the strong-scented oolong tea (Guo, Ho, Schwab, & Wan, 2021), and it also impacts the chemical profile and stability of roasted tea (Wang, Huang, Cao, Wang, & Xu, 2020).

On the one hand, roasting can markedly impact the non-volatile substances, which are mainly taste contributor substances and involved to shape the specific flavor of roasted tea. The chemical structures of origin secondary metabolites in tea leaves are transformed after roasting. During this process, the main polyphenols flavan-3-ols are prone to epimerization, oxidative polymerization, and nucleophilic addition reaction, which subsequently reduce the bitterness and astringency of tea infusion (Fan, Shi, Nie, Zhao, Ye, & Liang, 2016). It’s reported that epimerization was an important structural transformation of flavan-3-ols in the roasting process of green tea (Mizukami, Sawai, & Yamaguchi, 2008). Flavonoid profiles could distinguish different roasted green teas (Zhu et al., 2021). Besides, roasting can promote the oxidative decomposition of catechins, aldehydes, alcohols, and other substances (Zhang, Ho, Zhou, Santos, Armstrong, & Granato, 2019). Meanwhile, sugars, amino acids, and other substances, would convert into volatile components through the Maillard reaction and caramelization reaction, which contributed a lot to tea aroma formation indirectly (Zhou et al., 2019).

On the other hand, roasting can also change the volatile substances directly, thereby affect the aroma of roasted tea. The roasting process can significantly increase the contents of total aldehydes, total ketones, total esters, total furans, total pyrroles, and pyrazines of tea (Zhu et al., 2021). The main characteristic aroma substances induced by roasting include pyrazines, pyrroles, and furans, with pyrazines being one of the major odorants contributing to the aroma of roasted tea (KAWAKAMI and YAMANISHI, 1999, Sasaki et al., 2017).

In this study, taking *Tieguanyin* oolong tea as the object, we explored the effects of roasting on the compositions of non-volatile and volatile components using multi-platform instrumental analysis. Combined with sensory evaluation about the taste, mouthfeel attributes and aroma of oolong tea processed with different roasting treatments, we studied the relationship between flavor attributes and chemical compositions. The results can help to comprehensively understand the influence of roasting technology on the flavor and chemical substances of oolong tea. It is of great significance to guide the processing and production of oolong tea with different qualities and demands.

## 2 Materials and methods

### 2.1 Materials

Acetonitrile (ACN, HPLC grade), methanol (MeOH, HPLC grade), and water were purchased from Thermo Fisher Scientific Co., Ltd. (Shanghai, China). (+)-Catechin (C, > 98%), (−)-gallocatechin (GC, > 98%), (−)-catechin gallate (CG, > 98%), (−)-gallocatechin gallate (GCG, > 98%), (−)-epicatechin (EC, > 98%), (−)-epigallocatechin (EGC, > 98%), (−)-epicatechin gallate (ECG, > 98%), (−)-epigallocatechin gallate (EGCG, > 98%), gallic acid (GA, > 98%), l-theanine (> 99%), caffeine (> 98%), and theobromine (THB, > 98%) were obtained from Yuanye Biotechnology Co., Ltd. (Shanghai, China). *N*-Alkanes (C_7_-C_40_) were obtained from Aladdin Bio-Chem Technology Co., Ltd (Shanghai, China). All other reagents were of analytical grade.

*Tieguanyin* oolong tea samples were obtained from Anxi Taoyuan Organic Tea Farm Co. Ltd. These tea samples were made from the same fresh leaves and under the traditional *Tieguanyin* oolong tea manufacturing process, including withering, Zuoqing (a unique technology for oolong tea), fixing, rolling and roasting. All the samples were treated with the same procedure, except for the last one (i.e., roasting). Among them, the samples coded BT1/3/5 were roasted at a low fire (105 °C) for 1/3/5 h respectively, whereas AHT was treated by an additional roasting at 130 °C for 1 h based on BT5 for aroma-enhancing. The brewing water was from Hangzhou Wahaha Group Co., Ltd. (Hangzhou, China), a type of pure water same as what we used in previously studies (Cao et al., 2021, Xu et al., 2017, Xu et al., 2018, Xu et al., 2018).

### 2.2 Preparation of tea infusions

**For sensory evaluation and routine analysis.** Each tea sample of 5.0 g was brewed with 110 mL of boiling water three times in succession at room temperature (RT, 25 ± 2 °C), according to the method for oolong tea brewing in a covered bowl, as described in GB/T 23776–2018 (Administration of Quality Supervision & China, 2018).

**For non-targeted analysis.** The dry tea leaves were grounded into powder and then passed through a 100-mesh sieve. After that, 100 mg of tea powder was weighed and placed into a test tube with a glass stopper, and 5 mL of methanol was added. An ultrasonic water bath (40 °C, 20 min) was used for extraction. After the extraction was complete, the extracted solution and tea residues were transferred into a 10 mL volumetric flask, and methanol was added to reach constant volume. After standing for 10 min, a 1 mL aliquot of the upper layer was filtered through a 0.22 μm Millipore filter into the sample vials for injection analysis.

### 2.3 Sensory evaluation

The panel for sensory evaluation was composed of nine qualified panelists (five men, four women, 25 – 48 years old), all of whom were from the Tea Research Institute of the Chinese Academy of Agricultural Sciences and had achieved certificates for tea quality evaluation from the Tea Scientific Society of China. Sensory evaluation about flavor attributes, including taste, mouthfeel, and aroma, of oolong tea infusion was conducted for three times in different days at RT. The attributes for taste and mouthfeel included bitterness, astringency, and sweet aftertaste, whereas for aroma, there were “floral” (fresh, orchid-floral), “sweet” (fruity-sweet), and “roasted” (nutty, smoky). A 10-point scale (Xu et al., 2017) was used for grading the six flavor attributes, in which 0 – 2 was “very weak”, 2 – 4 “weak”, 4 – 6 “neutral”, 6 – 8 “strong”, and 8 – 10 “very strong”.

### 2.4 Analysis of color difference of tea infusions

Color difference of tea infusions was determined using a CM-3500d spectrophotometer (Konica Minolta (China) Investment Ltd., Shanghai, China) (Mao et al., 2021). The CIE L*a*b* system was adopted to describe the color of tea infusions. L* indicates the change in lightness from black (0) to white (1 0 0). The values of a* and b* indicate red (+a*)–green (−a*) and yellow (+b*)–blue (−b*), respectively. Distilled water was the control. Each sample was analyzed in triplicate.

### 2.5 Analysis of non-volatile substances of tea infusions

**Analysis of total soluble solids.** The content of total soluble solids in tea infusions were measured using a refractometer (RX-007α; Atago, Japan) after a zero calibration with distilled water.

**HPLC analysis.** HPLC was mainly used for the analysis of some targeted components, such as GA, GC, EGC, C, THB, EC, EGCG, GCG, ECG, Caffeine, and l-theanine. The HPLC system was equipped with an infinity binary pump, an autosampler, a column thermostat, and a diode array detector (Agilent Technologies, Santa Clara, CA, USA). The detailed detection method was referenced from a published article (Chen, Zhou, Meng, Zhang, Zhang, & Zhang, 2018). An Agilent Zorbax SB-Aq C_18_ column (250 × 4.6 mm i.d., 5 μm) equipped with a C18 guard column (Thermo Fisher Scientific, Waltham, MA, USA) was used for separation of analytes. The column temperature was set at 30 °C. The mobile phase A was 0.2% FA/H_2_O, and phase B was MeOH. The elution gradient started with 5% phase B (B), which was increased to 20% B at 5 min, increased to 25% B at 18 min, further increased to 42% B at 25 min and kept for 7 min, and finally increased to 100% B at 40 min. The total run time was 40 min. The flow rate was 1.0 mL/min. The injection volume was 5 μL. The detection wavelength was set at 278 nm.

**Non-targeted analysis based on UHPLC-HRMS.** An Agilent ultrahigh-performance liquid chromatography 6545 tandem Quadrupole time-of-flight mass spectrometer system (UHPLC-Q-TOF-MS, Agilent Technologies) was used for non-targeted analysis of tea samples. The parameters for UHPLC were set as follows. An Acquity UPLC Sheid RP-18 column (2.1 × 50 mm, 1.7 μm) was used for separation of analytes. The column temperature was set at 30 °C. The mobile phases A and B were H_2_O with 0.1% FA and ACN, respectively. The elution gradient was as follows: 0–5 min, 5–15% phase B (B); 5–8 min, 15–30% B; 8–13 min, 30% B; 13–23 min, 30–88% B; 23–28 min, 88–93% B; 28–30 min, 93% B; 30–33 min, 93–95% B; 33–35 min, 5% B. The flow rate was 0.3 mL/min. The injection volume was 2 μL. The full scan range of the UV detector was 190–400 nm, and the detection wavelength was 278 nm.

Data acquisition of mass spectrometry was based on the negative ion mode. The spray voltage was 3.5 kV. The flow rates of the dry gas and sheath gas were 8 and 11 L/min, respectively. The temperatures of the dry gas and sheath gas were 320 and 350 °C, respectively. The scan range for the mass-to-charge (*m*/*z*) ratio was 100–1500.

### 2.6 Analysis of volatile substances of oolong tea

Solid-phase microextraction (SPME, Supelco, Bellefonte, PA, USA) was used for extraction of volatiles in tea. The fused silica fiber used in SPME was coated with 50/30 μm of divinylbenzene/carboxen/polydimethylsiloxane. Before extraction, we performed 10 min pretreatment of the fiber in the gas chromatograph injection port at 250 °C in order to remove remaining volatiles from the fiber. Dry tea leaves (0.5 g) were weighed and placed in a 20 mL sealed glass vial. Subsequently, 5 mL of boiling water and 10 μL of internal standard solution (ethyl caprate, 10 mg/mL) were added. After 5 min of equilibration and stabilization, the vial was put into the thermostatic water bath at 60 °C for 60 min to adsorb the volatile components using SPME fiber. Subsequently, the volatiles were desorbed at the injector (250 °C) for 5 min.

The volatiles were analyzed by gas chromatography (GC) using an Agilent 6890 interfaced with an Agilent HP 5973 MSD ion trap mass spectrometer (Wilmington, DE, USA). A DB-5MS capillary column (30 m × 250 μm × 0.25 μm) was used for the GC separation. The injection temperature was 250 °C. The carrier gas was helium (99.999%), and the column flow rate was 1.0 mL/min. The split ratio was 15:1. The column temperature was 40 °C for the first 2 min. This was increased at 2 °C/min to 85 °C for 2 min, increased at 2.5 °C/min to 180 °C for 2 min, and then increased at 10 °C/min to 230 °C for 2 min. The ionization energy of the electron impact model in MS analysis was set at 70 eV. The temperature of the ion source was adjusted to 230 °C. The mass scan range was set to 40 – 400 *m*/*z*. Each sample was analyzed in triplicate. Agilent MassHunter Workstation Unknowns Analysis was used for compound identification based on the National Institute of Standards and Technology Library (NIST) and the retention index (Kovats index) of *n*-alkanes (C_7_-C_40_).

### 2.7 Statistical analysis

Data from scoring and measuring were presented as mean (in triplicate) ± standard deviation (SD). The significant differences were conducted by T-test between two groups, and one-way analysis of the variance (ANOVA) followed by Duncan test among three or more groups, using SAS software (Version 9.4, SAS Institute Inc., Cary, NC). SIMCA-P 14.1 software (Umetric, Umea, Sweden) was used for Partial least squares discriminant analysis (PLS-DA), and MultiExperiment Viewer software (version 4.7.4, Boston, MA) for heat-map analysis. Figures were plotted in GraphPad Prism (Version 9.00, GraphPad Software Inc., San Diego, CA).

## 3 Results and discussion

### 3.1 Effects of roasting on the sensory qualities of *Tieguanyin* oolong tea

**Fig. 1:**
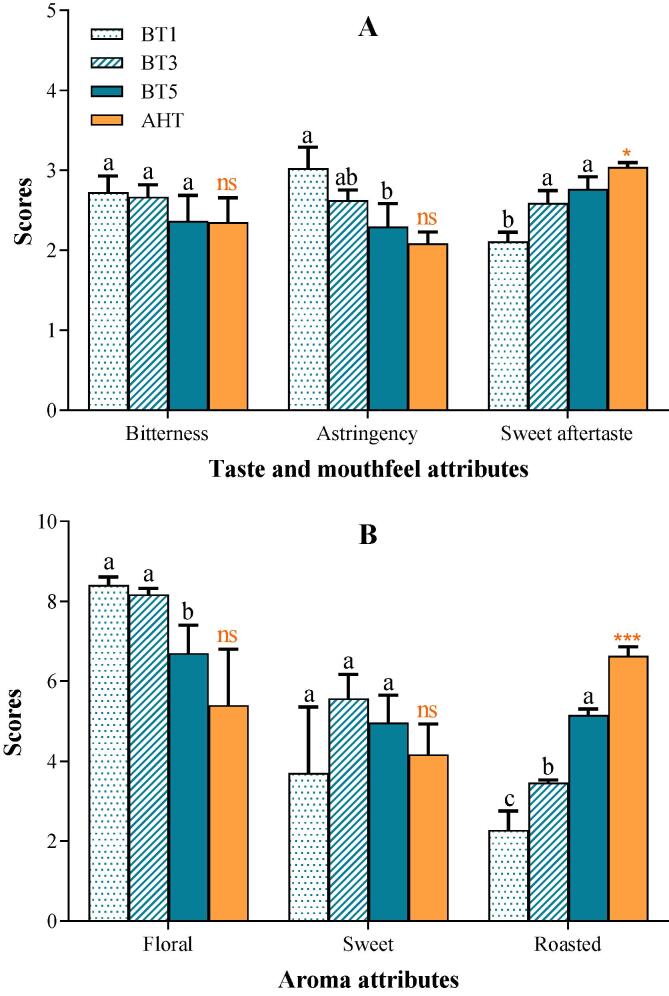
**Taste and mouthfeel, and aroma attributes of oolong tea** BT1/3/5 represent the oolong tea samples roasted for 1/3/5 h respectively; AHT represent the oolong tea sample after the aroma-enhancing treatment. ^a, b, c, d^ Different letters above the column indicate significant differences between different roasting time (*p* < 0.05); ns means no significant differences between the BT5 & AHT, * means *p* < 0.05, ^**^ means *p* < 0.01, ^***^ means *p* < 0.001.

The flavor characteristics of *Tieguanyin* oolong tea processed with different roasting treatments showed differences with varying degrees (Fig. 1). From the standpoint of the taste and mouthfeel attributes, the astringency decreased and sweet aftertaste increased significantly (*p* < 0.05), but the bitterness was stable with prolonging roasting time (Fig. 1A). For the aroma of traditional *Tieguanyin* oolong tea, fresh floral fragrance was dominant. Additional roasting treatment is able to enrich its aroma, producing caramelized, roasted incense or burnt scent, etc. (Huang & Sun, 2015). In this study, with the increase of roasting time, floral aroma showed a decreasing trend, whereas the roasted aroma increasing (Fig. 1B). The aroma-enhancing treatment (AHT) here is also a roasting treatment in fact, but with a higher temperature (130 °C). It aimed to develop and fix the qualities during the processing of oolong tea. Our results suggested that the effects of AHT on sensory attributes of *Tieguanyin* oolong tea were similar to that of roasting treatment mentioned above, but much more intensive.

**Fig. 2:**
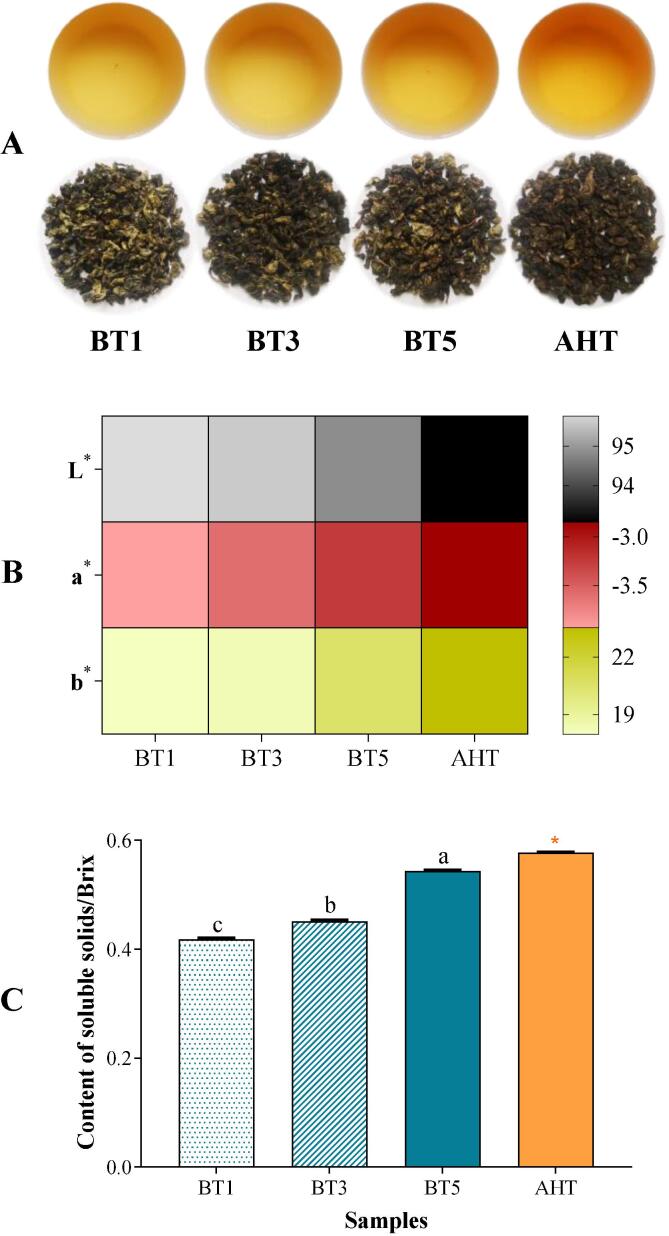
**The appearance of oolong tea & the contents of soluble solids in tea infusion** A: the pictures of tea leaves and infusions; B: the chromatic aberration of tea infusions; C: the contents of soluble solids in tea infusion. BT1/3/5 represent the oolong tea samples roasted for 1/3/5 h respectively; AHT represent the oolong tea sample after the aroma-enhancing treatment. ^a, b, c, d^ Different letters above the column indicate significant differences between different roasting time (*p* < 0.05); ns means no significant differences between the BT5 & AHT, * means *p* < 0.05, ^**^ means *p* < 0.01, ^***^ means *p* < 0.001.

In addition, the appearance color of tea leaves and infusions also changed a lot after roasting treatment or AHT (Fig. 2 A&B). With the deepening of roasting, the color of tea leaves and infusions darken gradually. The results of chromatic aberration in Fig. 2 B showed that there were significant correlations between the roasting time with L* (r = -0.953, *p* < 0.001), a* (r = 0.999, *p* < 0.001) and b* (r = 0.917, *p* < 0.001). It suggested that the brightness and greenness of oolong tea infusion would reduce, whereas yellowness raise, in proportion as roasting time increase. Wang et al. (2020) also found that tea infusion color changed regularly with roasting time.

### 3.2 Effects of roasting on non-volatile components of *Tieguanyin* oolong tea

#### 3.2.1 The content change of total soluble solids in tea infusion

The term of total soluble solids refers to the all substances dissolved in the tea infusion (Xiao, Zhang, Fan, & Han, 2017). Within certain range, higher content of soluble solids in tea infusion usually brings about a richer and fuller mouthfeel. In this study, the content of soluble solids increased proportionally as the roasting time (Fig. 2C).

#### 3.2.2 Effects of roasting on main taste components in the tea infusion

Using the targeted analysis with HPLC, we quantitatively detected some main taste compounds, including GA, GC, EGC, C, THB, EC, EGCG, GCG, ECG, caffeine, and l-theanine, shown as Table 1. The results indicated that the content of GA, non-epicatechins (except for C) and l-theanine increased with roasting time, while *epi*-catechins (except for ECG and EGCG) decreased. It was reported that GA had obvious characteristics of sweet aftertaste (Cao et al., 2019), which confirmed the results in Fig. 1A, i.e., sweet aftertaste of oolong tea infusion increased with roasting degree. EGCG and caffeine are the key components contributed to the bitterness of tea infusion (Xu, Zhang, Chen, Wang, Du, & Yin, 2018). In this study, their contents both remained basically unchanged, being consistent with the results in Fig. 1A as well. The AHT could intensify such effects of roasting time on all the components, with the exception of l-theanine. The content of l-theanine increased with the roasting time, but dropped sharply after AHT. Perhaps, it could be attributed to the condensation reaction of l-theanine with other substances under higher temperature (Guo et al., 2018, Zhou et al., 2019).

**Table 1:** The main taste components of oolong tea.

Chemicals	Content (mg/g)			
	BT1	BT3	BT5	AHT
GA	0.227 ± 0.004^c^	0.252 ± 0.011^b^	0.282 ± 0.003^a^	0.392 ± 0.022*
C	0.714 ± 0.002^a^	0.727 ± 0.008^a^	0.722 ± 0.013^a^	0.747 ± 0.033 ^ns^
GC	2.673 ± 0.143^c^	3.156 ± 0.078^b^	3.376 ± 0.060^a^	4.300 ± 0.095^***^
GCG	0.479 ± 0.010^b^	0.502 ± 0.044^ab^	0.539 ± 0.005^a^	0.854 ± 0.054^**^
EC	7.221 ± 0.174^a^	7.073 ± 0.356^a^	6.829 ± 0.148^a^	6.138 ± 0.148^**^
EGC	35.690 ± 0.049^a^	35.431 ± 1.031^a^	33.957 ± 0.167^b^	30.365 ± 0.427^***^
ECG	11.802 ± 0.671^a^	12.276 ± 0.420^a^	11.692 ± 0.116^a^	11.762 ± 0.340 ^ns^
EGCG	55.385 ± 0.237^ab^	56.239 ± 1.611^a^	53.441 ± 0.641^b^	52.325 ± 0.886 ^ns^
THB	0.170 ± 0.004^a^	0.173 ± 0.011^a^	0.184 ± 0.005^a^	0.174 ± 0.004 ^ns^
Caffeine	24.637 ± 0.062^a^	25.506 ± 0.757^a^	25.222 ± 0.159^a^	25.363 ± 0.487 ^ns^
l-theanine	1.657 ± 0.558^b^	3.923 ± 1.460^a^	3.726 ± 0.039^a^	0.715 ± 0.016^***^

Data are means (±SD) of three replicates.

BT1/3/5 represented the oolong tea samples roasted for 1/3/5 h respectively; AHT represented the oolong tea sample after the aroma-enhancing treatment.

### 3.3 Non-targeted UHPLC-Q-TOF-MS analysis of *Tieguanyin* oolong tea

**Fig. 3:**
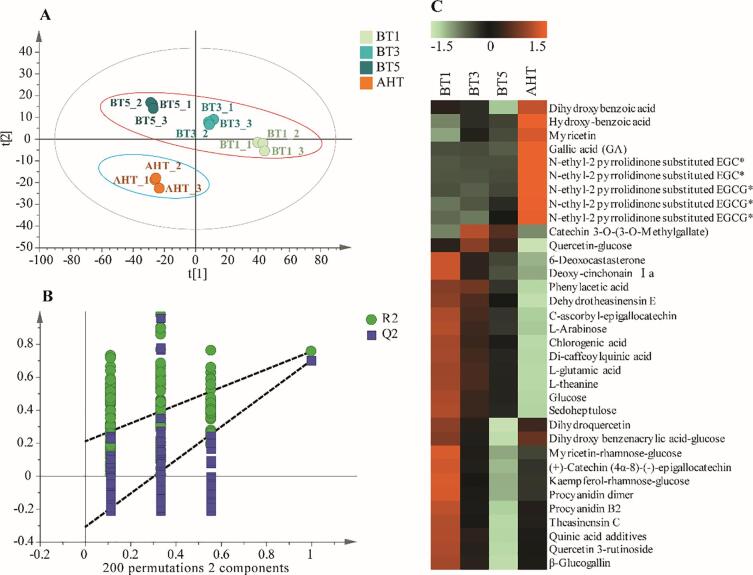
**The non-targeted metabolomics analysis based on LC-MS for oolong tea** A: the score plot of PLS-DA; B: overfitting prediction results; C: heat-map. BT1/3/5 represent the oolong tea samples roasted for 1/3/5 h respectively; AHT represent the oolong tea sample after the aroma-enhancing treatment.

Non-targeted analysis based on UHPLC-Q-TOF-MS was used to investigate the effects of roasting on the metabolic profile of *Tieguanyin* oolong tea, and the results are shown in Fig. 3.

It could be found that the oolong tea treated with AHT distinctly separated from those roasted for different time, from the score plot of PLS-DA (Fig. 3A). The tea samples roasted for 1/3/5 h also showed apparent separation and difference. These results implied the metabolic changes among oolong teas with different roasting treatments. The vector value of R^2^ (0.0, 0.208) and Q^2^ (0.0, −0.311) from 200 permutations, which indicated that this PLS-DA model was not overfitting (Fig. 3B). To explore metabolic changes during the roasting process, the key variables were screened out with Variable Importance in Projection (VIP) > 1. After the qualitative analysis of database searching, 34 substances were identified, and some substances were identified by their authentic standards. The significant changes of these substances were presented in the heat map (Fig. 3C). There were two changing trends for all the substances, of which one part decreased with the roasting time and the other increased.

The part of substances whose content correlated positively with the roasting time were mainly *N*-ethyl-2-pyrrolidinone-substituted flavan-3-ols. Within 5 h of roasting, the contents of these substances were very low or even none. However, their contents increased significantly after AHT. *N*-ethyl-2-pyrrolidinone-substituted flavan-3-ols had been identified in other types of tea. Wang et al. (2014) found 8-C *N*-ethyl-2-pyrrolidinone-substituted flavan-3-ols in ripened pu'er tea and suggested those can be used for the quality control and authentication of Chinese dark tea. 8-C *N*-ethyl-2-pyrrolidinone-substituted flavan-3-ols were also detected in the stored white tea, which correlated positively with the storage duration, and could be used as potential markers for long-term storage of white tea (Dai et al., 2018). In a study of large-leaf yellow tea, *N*-ethyl-2-pyrrolidinone-substituted flavan-3-ols were found to be produced from the condensation of flavan-3-ols and l-theanine, during the roasting process with high-temperature (Zhou et al., 2019). Our study confirmed that AHT with high temperature of 130 °C was the crucial factor for the formation of *N*-ethyl-2-pyrrolidinone-substituted flavan-3-ols in *Tieguanyin* oolong tea as well. Although some studies indicated that *N*-ethyl-2-pyrrolidinone-substituted flavan-3-ols had anti-inflammatory and antioxidant activity (Dai et al., 2020, Wang et al., 2014), studies on their sensory contribution and bioactive function are few.

The other part substances, decreasing with the roasting time, included flavonol glycosides (except myricetin), flavan-3-ols, amino acids, phenolic acids (except GA, dihydroxybenzoic acid, and hydroxy-benzoic acid), and saccharides. Zhu et al. (2021) reported that deep-roasting treatment could decrease the contents of most catechins and flavonol glycosides of green tea. The reduction of catechins and flavonol glycosides could contribute to the attenuation of astringency and bitterness (Chen, Kuo, Yang, Li, & Tzen, 2013). Amino acids have positive influences on tea infusion taste. l-theanine can relieve the bitterness and astringency, and enhance the umami and sweetness taste (Chen, Wang, Yuan, & He, 2021). l-glutamic acid can increase the umami taste (Zhang, Sun-Waterhouse, Su, & Zhao, 2019). During the roasting process, free amino acids and saccharides were vulnerable to non-enzymatic reactions under thermal action, such as Maillard reactions and Strecker degradation, which was beneficial for the formation of tea aroma (Jiang et al., 2020, Zhou et al., 2019).

### 3.4 Effects of roasting on volatile substances of *Tieguanyin* oolong tea

**Fig. 4:**
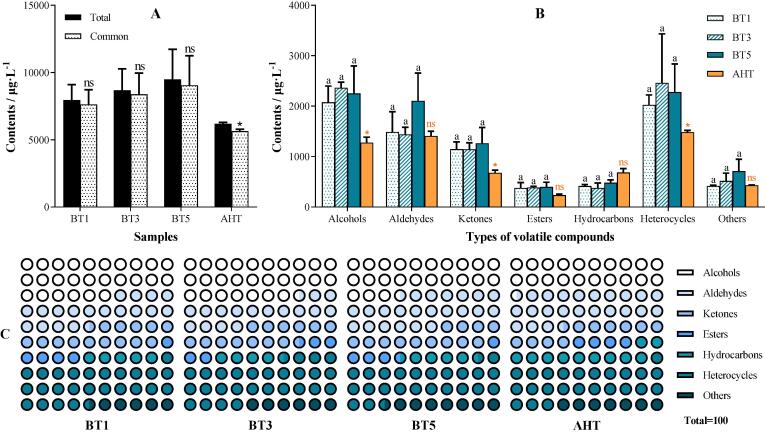
**The volatile chemicals in oolong tea** A: the contents of total and common (existing in all the samples) volatile chemicals; B & C: the content and percent of each type of volatile chemicals. BT1/3/5 represent the oolong tea samples roasted for 1/3/5 h respectively; AHT represent the oolong tea sample after the aroma-enhancing treatment. ^a, b, c, d^ Different letters above the column indicate significant differences between different roasting time (*p* < 0.05); ns means no significant differences between the BT5 & AHT, * means *p* < 0.05, ^**^ means *p* < 0.01, ^***^ means *p* < 0.001.

GC–MS analysis was performed to investigate the changes in volatile aroma substances. Aroma substances of 96 were totally detected. There were 68, 70, 70, and 78 aroma substances were found in oolong tea samples of BT1/3/5 and AHT, respectively. Among these, 51 aroma substances existed in all the tea samples (recorded as Common), and 22 unique aroma substances in different treatments, namely, 4/2/2 substances in BT1/3/5, respectively, and 14 substances in AHT. It was clear that the contents of the common volatiles still accounted for the main body (Fig. 4A). The detected aroma substances included alcohols (11), aldehydes (17), ketones (19), esters (14), hydrocarbons (19), heterocycles (11), and others (5). In terms of the number of volatile substances, the main class of volatile aroma substances were ketones, hydrocarbons, aldehydes, and esters. However, heterocycles, alcohols, aldehydes, and ketones were the most abundant volatile substances when it came to their contents (Fig. 4B). It was found that the volatiles content of each class was almost unaffected by roasting time, for the lack of significant difference among the treatments of BT1/3/5 (*p* > 0.05). But AHT did exert quite influences, such as reducing significantly (*p* < 0.05) the contents of total alcohols, ketones, and heterocycles. The percent of volatile chemicals in Fig. 4C also showed the similar trends, i.e., AHT being different obviously from the other three treatments. These results testified that AHT with temperature of 130 °C for short time played a key role during oolong tea process, based on the aroma formation.

The main aroma substances of oolong tea included linalool and its oxides (sweet floral), indole (animal-like), (E)-nerolidol (floral), α-farnesene (floral), phenylacetaldehyde (floral), hexanal (green grassy), and jasmone (jasmine floral) (Zheng, Li, Xiang, & Liang, 2016). Among these, (E)-nerolidol, α-farnesene, and indole were the main aroma substances for *Tieguanyin* tea (Ji, Liu, Xu, Jiang, Chen, & Yin, 2016). In this study, indole and (E)-nerolidol were still the two volatile substances with the highest contents in oolong tea with different roasting treatments. The contents of these main aroma substances were put in Fig. S1. The results were still similar to what mentioned above. All these substances were not influenced (*p* > 0.05) by the roasting treatment of long time at lower temperature (105 °C), but reduced dramatically (*p* < 0.05) (except for linalool) by AHT of short time at higher temperature of 130 °C, especially the α-farnesene, which was even undetected in AHT.

After roasting, especially AHT, some new volatile substances formed, including *cis*-α,α,5-trimethyl-5-vinyltetrahydrofuran-2-methanol, 3,4-dehydro-β-ionone, *trans*-β-ocimene, β-terpinene, 2,5-dimethyl-pyrazine, 2-acetylpyrrol and 1-(2-furylmethyl)-1H-pyrrole, etc. Most of these aroma substances belonged to pyrazines, pyrroles, and furans. These substances were produced in the Maillard reaction and caramelization reaction of sugars, amino acids, and pectins during the roasting process (Ho et al., 2015, Zhu et al., 2021). For example, 2,5-dimethyl-pyrazine was detected in the model thermal reaction of d-glucose and l-theanine (Guo et al., 2018). All of these kinds of substances had obvious roasted aroma, and their contents were proportional to the roasting degree.

**Fig. 5:**
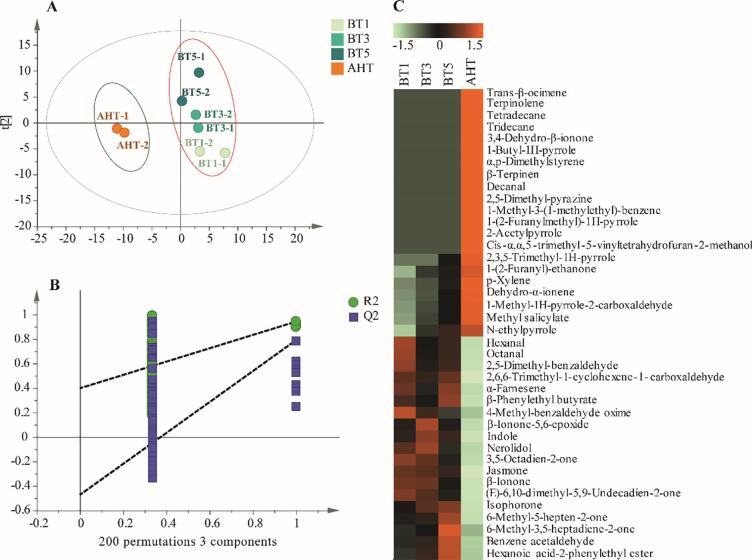
**The data analysis of volatile substances based on GC–MS for oolong tea** A: the score plot of PLS-DA; B: overfitting prediction results; C: heat-map. BT1/3/5 represent the oolong tea samples roasted for 1/3/5 h respectively; AHT represent the oolong tea sample after the aroma-enhancing treatment.

The analysis results of PLS-DA based on volatile substances in *Tieguanyin* oolong tea are showed as Fig. 5. The score plot in Fig. 5A indicated that all the samples could be classified into two groups, one was roasted for different time (BT1-5), and the other treated with aroma-enhancing (AHT), same as the results about non-volatile substances. The vector value of R^2^ (0.0, 0.398) and Q^2^ (0.0, −0.473) from 200 permutations suggested that this PLS-DA model was not overfitted (Fig. 5B). Then 40 volatile substances of VIP > 1 were screened out for further heat-map analysis (Fig. 5C) to explore metabolic changes during the roasting process. It can be seen that the contents of derivatives of pyrazines, pyrroles, and furans were extremely low in BT1-5, even undetected, but at a quite high level in the samples of AHT, such as 2,3,5-trimethyl-1H-pyrrole and *N*-ethylpyrrole (Fig. 5C), which was consistent with the results about the main aroma substances mentioned above. The contents of some alcohols, aldehydes, and ketones, including nerolidol, hexanal, and β-ionone decreased greatly after aroma-enhancing. The aldehydes and alcohols were easy to be oxidized and decomposed, and combined with amino acids to form the variety and roasted aroma of tea during the process (Ho et al., 2015, Hodge, 1953). Maybe, it is why the sample of AHT was with such a strong smell of roasting shown as Fig. 1B.

## 4 Conclusion

This study mainly focused on the effects of different roasting treatments on the flavor attributes and chemical compositions of *Tieguanyin* oolong tea using multiplatform analysis. It was found that the quality of oolong tea was closely related to the duration and temperature of the roasting procedure. Roasting treatment could shape the special flavor of oolong tea, like the formation of roasted aroma, and improve the overall mouthfeel, including reducing the astringency and increasing sweet aftertaste. With the extension of roasting time, the contents of amino acids, saccharides, alcohols, and aldehydes decreased, while the contents of GA and soluble solids increased. Thus, these trends were strengthened by AHT, which was the key process for flavor formation of strong-scented oolong tea. Besides, AHT could promote the formation of *N*-ethyl-2-pyrrolidinone-substituted flavan-3-ols, pyrazines, pyrroles, furans, and their derivatives. These results can give insight into the effects of roasting on the flavor and chemistry of *Tieguanyin* oolong tea.

## Declaration of Competing Interest

The authors declare that they have no known competing financial interests or personal relationships that could have appeared to influence the work reported in this paper.
