# Supplementary material for: Sensory and chemical characteristics of Tieguanyin oolong tea after roasting
Source: Food Chem X. 2021 Dec 2;12:100178. doi: 10.1016/j.fochx.2021.100178 (PMC8651997; doi:10.1016/j.fochx.2021.100178)
Supplement: Supplementary data 1 [file mmc1.docx]

**Sensory and chemical characteristics of *Tieguanyin* oolong tea after roasting**

Qing-Qing Cao ^a, b, 1^, Yan-Qing Fu ^a, 1^, Jie-Qiong Wang ^a.b^, Liang Zhang ^c *^,

Fang Wang ^a^, Jun-Feng Yin ^a^, Yong-Quan Xu ^a **^

*^a^ Tea Research Institute* *Chinese Academy of Agricultural Sciences, Key Laboratory of Tea Biology and Resources Utilization, Ministry of Agriculture, 9 South Meiling Road, Hangzhou 310008, China.*

*^b^ Graduate School of Chinese Academy of Agricultural Sciences, Beijing 100081, China*

^c^ *State Key Laboratory of Tea Plant Biology and Utilization, Anhui Agricultural University, Hefei, China*

^1^ These authors contributed equally to this work.

**Corresponding Authors:**

^**^Tel: +86-571-86650594, Fax: +86 571 86650056. Email: [yqx33@126.com](mailto:yqx33@126.com) (Y.-Q. Xu)

^*^ Email: [zhli2091@sina.com](mailto:zhli2091@sina.com) (L. Zhang)

**Fig. S1. The content changes of some volatile substances in oolong tea roasting different time**

BT1/3/5 represent the oolong tea samples roasted for 1/3/5 h respectively; AHT represent the oolong tea sample after the aroma-enhancing treatment.

^a, b, c, d^ Different letters above the column indicate significant differences between different roasting time (*p* < 0.05); ns means no significant differences between the BT5 & AHT, ^*^ means *p* < 0.05, ^**^ means *p* < 0.01, ^***^ means *p* < 0.001.
